# High prevalence of vitamin D deficiency among women of child-bearing age in Lahore Pakistan, associating with lack of sun exposure and illiteracy

**DOI:** 10.1186/s12905-015-0242-x

**Published:** 2015-10-12

**Authors:** Kashaf Junaid, Abdul Rehman, David A. Jolliffe, Kristie Wood, Adrian R. Martineau

**Affiliations:** Department of Microbiology and Molecular Genetics, University of the Punjab, Quaid-e-Azam Campus, Lahore, 5400 Pakistan; Centre for Primary Care and Public Health, Barts and The London School of Medicine and Dentistry, Queen Mary University of London, London, E1 2AB UK; Genome Centre, Barts and the London School of Medicine and Dentistry, Queen Mary University of London, London, EC1M 6BQ UK

**Keywords:** Vitamin D receptor, Hypovitaminosis D, Cross sectional study

## Abstract

**Background:**

Vitamin D status is a key determinant of maternal and neonatal health. Deficiency has been reported to be common in Pakistani women, but information regarding environmental and genetic determinants of vitamin D status is lacking in this population.

**Methods:**

We conducted a cross-sectional study among three groups of healthy women living in Lahore, Pakistan: university students, students or employees of Medrasas or Islamic Institutes, and employees working in office, hospital or domestic settings. Multivariate analysis was performed to identify environmental and genetic determinants of vitamin D status: polymorphisms in genes encoding the vitamin D receptor, vitamin D 25-hydroxylase enzyme CYP2R1 and vitamin D binding protein [DBP] were investigated. We also conducted analyses to identify determinants of body ache and bone pain in this population, and to determine the sensitivity and specificity of testing for hypocalcaemia and raised serum alkaline phosphatase to screen for vitamin D deficiency.

**Results:**

Of 215 participants, 156 (73 %) were vitamin D deficient (serum 25[OH]D <50 nmol/L). Risk of vitamin D deficiency was independently associated with illiteracy (adjusted OR 4.0, 95 % CI 1.03–15.52, *P* = 0.04), <30 min sun exposure per day (adjusted OR 2.13, 95 % CI 1.08–4.19, *P* = 0.02), sampling in January to March (adjusted OR 2.38, 95 % CI 1.20–4.70), *P* = 0.01) and lack of regular intake of multivitamins (adjusted OR 2.61, 95 % CI 1.32–5.16, *p* = 0.005). Participants with the GG genotype of the rs4588 polymorphism in the gene encoding vitamin D binding protein tended to have lower 25(OH)D concentrations than those with GT/TT genotypes (95 % CI for difference 22.7 to −0.13 nmol/L, *P* = 0.053). Vitamin D deficiency was independently associated with increased risk of body ache or bone pain (adjusted OR 4.43, 95 % CI 2.07 to 9.49, *P* = 0.001). Hypocalcaemia (serum calcium concentration ≤9.5 mg/dL) and raised alkaline phosphatase concentration (≥280 IU/L) had low sensitivity and very low specificity for identification of vitamin D deficiency.

**Conclusion:**

Vitamin D deficiency is common among healthy women of child-bearing age in Lahore, Pakistan: illiteracy, decreased sun exposure and lack of multivitamin intake are risk factors.

## Background

Despite its sub-tropical location and sunny climate, vitamin D deficiency has been reported to be very common in Pakistan, especially among women: a recent study reported that 90 % of pre-menopausal females had serum 25-hydroxyvitamin D (25[OH] D) concentrations <50 nmol/L (20 ng/mL) [1]. A growing literature points to the importance of adequate maternal vitamin D status in protecting against a wide range of adverse obstetric and neonatal outcomes, including pre-term birth, fetal neurodevelopment and neonatal infection [2, 3]. Observational studies have reported associations between vitamin D deficiency and a wide range of clinical conditions including osteopenia, osteoporosis, body ache and bone pain, and several cancers, autoimmune diseases and infectious diseases [4]. Although vitamin D status may be influenced by environmental factors such as sun exposure and diet, variation in the genes encoding the vitamin D 25-hydroxylase enzyme CYP2R1, the vitamin D binding protein (DBP) and the vitamin D receptor (VDR) have also been reported to associate with risk of vitamin D deficiency [5, 6]. The relative contribution of environmental vs. genetic determinants of risk of vitamin D deficiency has not previously been described in Pakistani women.

We therefore conducted a cross-sectional study in 215 adult women living in three different settings in Lahore, Pakistan, to determine both environmental and genetic determinants of vitamin D deficiency in this population. We also conducted statistical analyses to determine whether low vitamin D status was independently associated with risk of body ache and bone pain in this population; and to establish the sensitivity and specificity of screening for vitamin D deficiency by testing for hypocalcaemia or raised serum alkaline phosphatase concentration.

## Methods

### Subject selection

Healthy adult females were screened for eligibility to participate in the study from January 2012 to July 2012 in Lahore, Pakistan. Three groups of women were sampled: students at the University of the Punjab Campus; students and staff at Madrasas or Islamic institutes; and employees working in offices, hospitals and domestic settings (maids). Exclusion criteria were: age <15 or >50 years; presence of symptoms or signs of active infectious disease or chronic illness; abnormal renal/liver function (serum glutamate pyruvate transaminase [SGPT] ≥ 18 IU/L, serum glutamate oxaloacetate [SGOT] ≥16 IU/L, serum urea concentration ≥7.5 mmol/L, serum alkaline phosphatase concentration ≥280 IU/L); presence of Hepatitis B surface antigen (HBSAg) or antibodies to Hepatitis C virus (HCV) or Human Immunodeficiency Virus (HIV); and pregnancy.

A detailed interview was conducted to gather details about participants lifestyle and social status; questions were asked about participants’ monthly income (subjects with monthly income <10,000 PKR were classified as having lower incomes, subjects with monthly income 10,000–20,000 PKR were considered to have intermediate incomes and subjects with monthly income >20,000 PKR were considered to have higher incomes), marital status, preference of food items (we asked a list of various items e.g. fish, chicken, mutton, vegetables, wheat, rice burger, pizza and rice and from that data we subdivided into two groups – ‘homemade food’ or ‘junk food’), milk intake, and their hours of sun exposure sufficient to induce cutaneous vitamin D synthesis. Participants were asked if they were exposed to sun or had outdoor activity in between 10:00 AM and 3:00 PM, and whether or not they used any sun protection factor (SPF) product or a veil while they were outside. Hours spent outside with use of SPF products or veiling were not counted towards total hours of sun exposure. Participants were also asked whether they used any multivitamin supplements. A clinical history was also taken, to identify potential participants with fever, cough, active infectious disease and a diagnosis of any chronic illness. We also enquired about history of body ache/pain, by asking about the presence of any of the following symptoms: lower backache or backache after long periods standing, morning stiffness, and joint pain. Participants were then weighed, and their height was measured: body mass index (BMI) was calculated as weight (kg)/[height (m)^2^]. A general clinical examination was performed to exclude subjects with jaundice or pedal edema.

From the selected subjects five ml of blood was drawn from the median cubital vein 2 ml was immediately transferred into EDTA coated vials and used for genomic study while 3 ml of whole blood was transferred into serum vials; serum samples were used for biochemical analysis. The study was approved by the ethics committee of the School of Biological Sciences, University of Punjab (ref SBS 873–12) and informed consent was obtained from all subjects prior to participation, parental consent was taken from those aged below 16 years; this was recorded in writing for subjects who were able to read and write.

### Screening

Serum of each subject was screened for HBsAg and antibodies to HCV, HIV using ABON kits (Abon Biopharm, Hangzhou); manufacturers’ instructions were followed and any participants found positive for these tests were excluded from this study. For renal and liver function tests, serum urea, alkaline phosphate, SGPT and SGOT were done using Bio Bas kits (SPINERACT, S.A. Spain).

### Serum 25(OHD assay

Serum 25(OH)D concentrations was measured by IDS enzyme immunoassay (Immunodiagnostic Systems, Boldon, UK). The calibrators and controls were provided with each kit was run in duplicate. In this study we classified vitamin D deficiency <50 nmol/L based on the previous definition of vitamin D deficiency as per the Endocrine Society Clinical Practice Guideline [7]. The inter-assay CV for this assay was 15 %.

### Serum calcium and alkaline phosphatase

Serum calcium and alkaline phosphatase were measured by a photometric method using the BioBas kit (SPINREACT, S.A. Spain). Calibrator for serum calcium and alkaline phosphatase was run along with samples.

### Genotyping

DNA was extracted from whole blood using the protocol published by Iranpur et al. [8] and quantified using a nanodrop spectrophotometer. A 5–10 ng of DNA sample was used for TaqMan allelic discrimination assays. Pre-developed assays were used to type polymorphisms in the genes encoding VDR (rs2228570 [FokI], rs731236 [TaqI] and rs1544410 [BsmI]), CYP2R1 (rs2060793, rs10500804 and rs10766197) and DBP (rs7041 [HaeIII] and rs4588 [StyI]). Alleles at all loci conformed to the Hardy-Weinberg equilibrium.

### Sample size and statistical analysis

This study was powered to estimate prevalence of vitamin D deficiency in the healthy female population with a 5 % error margin at the 95 % confidence level, using algorithms published by Lwanga and Lemishow [9]; assuming an expected prevalence for vitamin D deficiency of 80 % in Lahore [10], we calculated that a total of *n* = 247 participants would need to be recruited.

The data were analyzed by using statistical package for social sciences version 20 (SPSSv.20).

Descriptive statistics were used to calculate proportions and percentages for each categorical variable used in univariate analysis. Adjusted odds ratios for potential determinants of vitamin D status and bone pain were calculated by logistic regression analysis; vitamin D deficiency was defined as serum 25(OH)D concentration <50 nmol/L. All correlates of vitamin D status with *p* value less than 0.1 on univariate analysis were included in the multivariate model. Associations between genotype and serum 25(OH)D concentrations were analyzed by unpaired t tests. In order to explore the accuracy with which hypocalcaemia or increased serum alkaline phosphatase concentration could be used to identify participants with vitamin D deficiency, we calculated the sensitivity and specificity of hypocalcaemia (≤9.5 mg/dL) and raised alkaline phosphatase concentration (≥280 IU/L) for identification of serum 25(OH)D concentration <50 nmol/L in study participants.

## Results

### Participants enrolment

Three hundred adult females were enrolled in the study in Lahore, Pakistan. Of these, 36 were excluded due to positive tests for hepatitis viral infection (19 were positive for HBsAg, 9 positive for anti-HCV antibody and 8 were positive for both HBsAg and anti-HCV). An additional 49 screened participants were excluded later due to deranged liver and renal function tests (for cut-off values, see Methods). The final analysis was performed on data from 215 subjects who fulfilled all study eligibility criteria.

### Demographic characteristics

Table 1 presents the demographic characteristics of these 215 subjects. Ninety-two (43 %) samples were from university students, 56 (26 %) were from students and staff of religious institutes and 67 (31 %) were from working females. The mean age was 28.4 years (standard deviation 7.2), and the age range was from 15 to 45 years. Of two hundred and fifteen participants, 134 (62 %) were aged between 15–30 years and 81 (38 %) were aged between 31–45 years. Most of the participants 150, (70 %) were from Punjab; 65 (30 %) were from other provinces and were residing in Lahore. One hundred and twenty-six participants (59 %) had BMI <25 kg/m^2^, 107 (50 %) of them preferred homemade food and 92 (43 %) of them had milk in their daily diet. Of the total female subjects 134 (62 %) had sun exposure time >30 min per day.

**Table 1:** Demographic characteristics of subjects included in this study (*n* = 215)

Characteristics		N (%)
Age	15–30 years	134 (62 %)
	31–45 Years	81 (38 %)
	Mean age, years (S.D)	28.36 (7.24)
	Range, years	15–45
Settings	University students	92 (43 %)
	Employed	56 (26 %)
	Religious settings	67 (31 %)
Month of recruitment	January-March	104 (48 %)
	April - July	111 (52 %)
Marital Status	Single	84 (39 %)
	Married	131 (61 %)
Province/place of origin	Punjabi	150 (70 %)
	NWFP^a^	32 (15 %)
	Gilgit Baltistan	33 (15 %)
Education	Illiterate	27 (13 %)
	5–10 years of education	91 (42 %)
	11–16 years of education	97 (45 %)
Income^b^	Lower Income	77 (36 %)
	Intermediate income	87 (40 %)
	Higher income	51 (24 %)
Body mass index	BM1 < 25 kg/m^2^	126 (59 %)
	BMI ≥ 25 kg/m^2^	89 (41 %)
Preferred food item^c^	Homemade food	108 (50 %)
	Junk food	107 (50 %)
Milk Intake	Yes	92 (43 %)
	No	123 (57 %)
Sun exposure time/day	≤30 min	116 (54 %)
	>30 min	99 (46 %)
Body area exposed	Veil	114 (47 %)
	No veil	101 (53 %)
Taking Multivitamin	Yes	78 (36 %)
	No	137 (64 %)
25-hydroxyvitamin D level (nmol/l)	Mean (S.D)	40.43 (34.45)
	<25 nmol/l	92 (43 %)
	25–49.9 nmol/l	64 (30 %)
	50–74.9 nmol/l	37 (17 %)
	≥75 nmol/	22 (10 %)

^a^NWFP, North West Frontier Province

^b^Socioeconomic status was classified depending upon monthly income; Lower class: monthly income <10,000PKR, intermediate income: monthly income 10–20,000PKR, higher income: monthly income >20,000PKR

^c^Preference of food item was classified depending what they eat most regularly either junk food or if they prefer to make a food at home

The mean serum concentration of 25(OH)D in all participants was 40.4 nmol/L [SD] 34.5; 92 participants (43 %) had a 25(OH) D level lower then 25 nmol/L, 64 (30 %) had 25(OH)D levels of 25 to 49.9 nmol/L, 37 (17 %) had 25(OH)D levels from 50–74.9 nmol/L and only 22 (10 %) had 25(OH)D levels ≥75 nmol/L.

### Social determinants of vitamin D status

Table 2 presents demographic, social and behavioral factors that were associated with risk of vitamin D deficiency in study participants. Multivariate analysis revealed independent associations between vitamin D deficiency and a) illiteracy (adjusted OR 4.0, 95 % CI 1.03–15.52, *P* = 0.04), b) sun exposure time less than 30 min per day (adjusted OR 2.13, 95 % CI 1.08–4.19, *P* = 0.02), c) sampling season January to March (adjusted OR 2.38, 95 % CI 1.20–4.70), *P* = 0.01) and d) lack of regular intake of multivitamins (adjusted OR 2.61, 95 % CI 1.32–5.6, *P* = 0.005). No significant independent relationship was found between risk of vitamin D deficiency and age, recruitment setting, marital status, monthly income, preferred food items, body exposure, milk intake, BMI or state of origin.

**Table 2:** Demographic, social and behavioral determinants of vitamin D status in healthy adult females (*n* = 215)

Determinants		25(OH) D < 50 nmol/l	Univariate analysis		Multivariate analysis	
			Odds ratio (95 % CI)	*p*-value	^d^Adjusted odds ratio (95 % CI)	*p*-value
Age	15–30 years	90/134 (67 %)	Ref	-	Ref	-
	31–45 years	66/81 (81 %)	2.15 (1.10–4.18)	0.02	1.63 (0.74–3.58)	0.22
Settings	University students	60/92 (65 %)	1.7 (0.83–3.44)	0.14		
	Religious institutes	45/56 (80 %)	0.78 (0.32–1.92)	0.60		
	Employed	51/67 (76 %)	Ref	-		
Month of recruitment	January-March^a^	85/104 (81 %)	2.51 (1.34–4.73)	0.004	2.38 (1.20–4.70)	0.01
	April-July^b^	71/111 (64 %)	Ref	-	Ref	-
Marital status	Single	53/84 (63 %)	Ref	-	Ref	-
	Married	103/131 (78 %)	2.15 (1.17–3.95)	0.01	1.49 (0.72–3.09)	0.27
Education	Illiterate	24/27 (89 %)	4.12 (1.15–14.71)	0.03	4.0 (1.03–15.52)	0.04
	5–10 years	68/91 (75 %)	2.70 (0.74–9.8)	0.13	2.71 (0.68–10.72)	0.15
	11–16 years	64/97 (66 %)	Ref	-	Ref	-
Income	Lower income	54/77 (70 %)	1.02 (0.47–2.21)	0.95		
	Intermediate income	66/87 (76 %)	0.70 (0.35–1.66)	0.56		
	Higher income	36/51 (70 %)	Ref	-		
Preferred food item	Homemade food	84/108 (78 %)	Ref	-	Ref	-
	Junk food	72/107 (67 %)	1.70 (0.92–13.12)	0.08	1.31 (0.67–2.57)	0.42
Body exposure	Full body covered/veil	70/114 (61 %)	1.60 (0.93–2.75)	0.31		
	Some parts exposed	86/101 (85 %)	Ref	-		
Sun exposure time/day	≤30 min	89/116 (77 %)	2.09 (1.14–43.85)	0.01	2.13 (1.08–4.19)	0.02
	>30 min	67/99 (68 %)	Ref	-	Ref	
Milk intake	Yes	61/92 (66 %)	Ref	-	Ref	-
	No	95/123 (77 %)	1.72 (0.94–3.15)	0.07	1.46 (0.74–2.88)	0.26
Province/place of origin	Punjabi	109/150 (73 %)	Ref	-		
	Others^c^	47/65 (72 %)	1.01 (0.53–1.95)	0.95		
BMI	<25 kg/m^2^	87/126 (69 %)	Ref	-		
	≥25 kg/m^2^	69/89 (77 %)	1.54 (0.82–2.8)	0.17		
Use of a multivitamin supplement	Yes	32/78 (41 %)	Ref	-	Ref	-
	No	124/137 (90 %)	2.56 (1.38–4.74)	0.002	2.61 (1.32–5.16)	0.005

*25(OH) D* 25- hydroxy vitamin D, *Ref* Reference Category

^a^Winter, samples recruited in January, February and March

^b^Summer, samples recruited in April, May, June and July

^c^Others, people from NWFP (North West Ffrontier Pakistan) and Gilgit Baltistan

^d^Adjusted for age, month of recruitment, marital status, education, preferred food item, sun exposure time/day, milk intake and regular intake of a multi vitamin supplement

**Table 3:** Genetic determinants of vitamin D status in healthy adult females

SNP	Genotype	Serum 25(OH) D <50 nmol/L N (%)	^a^OR (95 % CI)	*P* value
VDR, rs731236^a^	AA	53/65 (81 %)	Ref	-
	AG/GG	72/94 (77 %)	1.57 (0.65–3.78)	0.31
VDR, rs1544410^b^	CC	36/45 (80 %)	Ref	-
	CT/TT	92/118 (80 %)	1.72 (0.65–4.54)	0.26
VDR, rs2228570^c^	GG	85/107 (79 %)	Ref	-
	GA/AA	43/57 (75 %)	2.07 (0.84–5.09)	0.11
CYP2R1, rs10500804^d^	GG	33/44 (75 %)	Ref	-
	GT/ TT	90/115 (78 %)	0.76 (0.30–1.93)	0.57
CYP2R1, rs2060793^e^	GG	60/81 (74 %)	Ref	-
	GA /AA	66/79 (83 %)	0.57 (0.21–1.23)	0.13
Cyp2R1, rs10766197^f^	AA	28/38 (74 %)	Ref	-
	AG/ GG	98/124 (79 %)	0.83 (0.32–2.15)	0.70
DBP, rs7041^g^	AA	29/36 (80 %)	Ref	-
	AC/CC	96/124 (77 %)	1.16 (0.41–3.24)	0.77
DBP, rs4588^h^	GG	63/76 (83 %)	Ref	-
	GT /TT	65/88 (74 %)	2.21 (0.89–5.00)	0.09

^a^OR odd ratios adjusted for education, sun exposure time, month of recruitment and multivitamin supplementation; SNP, single nucleotide polymorphism, VDR, Vitamin D receptor; DBP, Vitamin D binding protein; CYP2R1, Vitamin D 25-hydroxylase

^a^ *n* = 159

^b^ *n* = 163

^c^ *n* = 164

^d^ *n* = 159

^e^ *n* = 160

^f^ *n* = 162

^g^ *n* = 160

^h^ *n* = 164

### Genetic determinants of vitamin D status

Of the eight SNP studied, none showed a statistically significant association with serum 25(OH) D concentration in the study population. However, rs4588 we observed a trend towards lower serum 25(OH)D concentrations in participants with the GG genotype, as compared with those with GT or TT genotypes (mean serum 25(OH) D concentration 32.6 vs. 43.9 nmol/L respectively; 95 % CI for difference, 22.70 to −0.13 nmol/L; *P* = 0.053) (Table 3).

### Determinants of bone pain/ body ache

Of the 111 women reporting bone pain/body ache, 58 (52.3 %) had serum 25(OH)D <25 nmol/L, 37 (33.3 %) had serum 25(OH)D ≥ 25 nmol/L and <50 nmol/L, and 16 (14.4 %) had 25(OH)D >50 nmol/L. Multivariate analysis revealed that risk of body ache or bone pain in the study population was independently associated with a) being married (adjusted OR 7.11, 95 % CI 3.44–14.70, *P* = <0.001), and b) vitamin D deficiency (adjusted OR 4.43, 95 %CI 2.07–9.49, *P* = 0.001) (Table 4).

**Table 4:** Determinants of body ache or bone pain in healthy adult females (*N* = 215)

Determinants		Presence of Bone Pain/ Body ache (*N* = 111)	Univariate analyses		Multivariate analyses	
			Odds ratio (95 % CI)	*p*-value	^a^Adjusted odds ratio (95 % CI)	*p*-value
Age	15–30 years	61/134 (45 %)	Ref	-	Ref	-
	31–45 years	50/81 (65 %)	1.93 (1.10–3.47)	0.02	0.76 (0.37–1.55)	0.46
Settings	University students	42/92 (455 %)	0.71 (0.36–1.32)	0.23		
	Religious institutes	32/56 (57 %)	1.11 (0.53–2.21)	0.83		
	Employed	37/67 (55 %)	Ref	-		
Marital status	Single	21/84 (25 %)	Ref	-	Ref	-
	Married	90/131 (70 %)	6.58 (3.55–12.20)	<0.001	7.11 (3.44–16.70)	<0.001
Education	Illiterate	17/27 (66 %)	1.24 (0.29–0.93)	0.03	0.57 (0.20–1.56)	0.27
	5–10 years	38/91 (44 %)	0.52 (0.26–1.96)	0.62	1.58 (0.56–4.48)	0.38
	11–16 years	56/97 (57 %)	Ref	-	Ref	-
Socioeconomic status	Lower class	38/77 (48 %)	0.86 (0.42–1.75)	0.70		
	Middle class	46/87 (55 %)	1.01 (0.50–2.01)	1.0		
	Upper class	27/51 (55 %)	Ref	-		
Preferred food item	Homemade food	63/108 (61 %)	Ref		Ref	
	Junk food	48/107 (44 %)	1.72 (1.0–2.95)	0.05	0.62 (0.33–1.18)	0.15
Milk intake	Yes	48/92 (53 %)	Ref			
	No	63/123 (52 %)	1.03 (0.60–1.70)	0.90		
BMI	<25 kg/m^2^	62/126 (49 %)	Ref			
	≥25 kg/m^2^	49/89 (57 %)	1.35 (0.80–2.20)	0.40		
Multivitamin use	Yes	39/78 (50 %)	Ref			
	No	72/137 (54 %)	1.10 (0.63–2.02)	0.70		
Serum 25(OH)D concentration	<50 nmol/l	95/156 (60 %)	4.18 (2.16–8.08)	0.001	4.43 (2.07–9.49)	<0.001
	≥50 nmol/l	16/59 (27 %)	Ref		Ref	

*BMI* body mass index

^a^Adjusted for age, marital status, education, preferred food item and vitamin D level

### Sensitivity and specificity of hypocalcaemia and raised serum alkaline phosphatase in identifying vitamin D deficiency

Hypocalcaemia (serum calcium concentration ≤9.5 mg/dL) had low sensitivity (77.6 %) and very low specificity (27.1 %) for identification of vitamin D deficiency (Table 5). Raised serum alkaline phosphatase concentration (≥280 IU/L) had low sensitivity (60.3 %) and very low specificity (18.6 %) for identification of vitamin D deficiency (Table 6).

**Table 5:** Sensitivity and specificity of hypocalcaemia for identification of vitamin D deficiency defined using the 50 nmol/L 25(OH)D threshold

Serum Calcium	25(OH)D <50 nmol/L	25(OH)D ≥50 nmol/L	Sensitivity	Specificity
≤9.5 mg/dl	121	43	77.56 %	27.12 %
>9.5 mg/dl	35	16

**Table 6:** Sensitivity and specificity of elevated serum alkaline phosphatase concentration for identification of vitamin D deficiency defined using the 50 nmol/L 25(OH)D threshold

Serum alkaline phosphatase	25(OH)D <50 nmol/L	25(OH)D ≥50 nmol/L	Sensitivity	Specificity
<280 IU/L	62	11	60.25 %	18.64 %
≥280 IU/L	94	48		

## Discussion

We report that inadequate vitamin D status is very common among healthy women of child bearing age living in Lahore, Pakistan: only 10 % of participants in our study had optimal vitamin D status (serum 25[OH]D ≥75 nmol/L), 73 % were vitamin D deficient (serum 25[OH] D <50 nmol/L) and 43 % were profoundly deficient (serum 25[OH]D <25 nmol/L). Risk of deficiency was independently associated with sampling in January - March, illiteracy, decreased sun exposure and lack of multivitamin use; we also found evidence that vitamin D binding protein genotype may be a determinant of vitamin D status in this population. Vitamin D deficiency was independently associated with increased risk of bone pain and body ache. The use of tests for hypocalcaemia or raised serum alkaline phosphatase concentration was neither sensitive nor specific for the identification of vitamin D deficiency. It has also been reported previously that serum calcium does not predict serum 25(OH)D levels [11].

Our finding of very high prevalence of inadequate vitamin D status is in keeping with those of other investigators, who have reported a low prevalence of optimal vitamin D status among women in Karachi, Pakistan, where one study has reported that 84 % of participants had serum 25(OH)D concentrations <75 nmol/L [12]; another has reported that 91 % of participants had serum concentrations <50 nmol/L [1]. Studies conducted elsewhere in Asia have reported similarly high rates of vitamin D deficiency among women [13–15]. Although we showed that vitamin D deficiency was associated with decreased sunlight exposure, we did not demonstrate any association between veiling and risk of vitamin D deficiency; this finding is consistent with that from a recent study in Bangladesh, which reported that vitamin D deficiency was common among women in that setting irrespective of veiling [13].

Our study has several strengths. The fact that we recruited participants in three different settings enhances generalizability of our findings. We also collected detailed information on potential determinants of vitamin D status, including genetic determinants. Our finding that the rs4588 (StyI) polymorphism in the vitamin D binding protein associates with serum 25(OH)D concentration is consistent with reports elsewhere in the literature [16]. DBP genotype has been reported to influence total 25(OH)D levels by other investigators [5]. This may reflect the influence of differing variants’ affinity for 25(OH)D on its half-life in the circulation, or differing concentrations of DBP between individuals having different DBP genotypes.

Our study also has some limitations. We did not record details of individual participants’ skin pigmentation. Neither did we record the vitamin D content of multivitamin preparations, or measure serum PTH concentrations. Our study was not formally powered to detect the influence of genetic variation in the vitamin D pathway on serum 25(OH) D concentrations; although we were powered to detect clinically significant genetic effects, it is possible that we lacked statistical power to detect smaller effect sizes of genetic determinants. An important clinical outcome of vitamin D deficiency is osteomalacia, which has been previously reported in females of the Hazara Division, Pakistan [17] but due to the ethical concerns we were unable to access bone biopsy samples of participants in our study and correlate histological findings with vitamin D deficiency.

## Conclusion

In conclusion, we demonstrate very high rates of vitamin D deficiency among healthy women living in Lahore, Pakistan, and show that use of a multivitamin tablet is protective. Our findings suggest that women in this setting who are not in a position to increase their sun exposure can significantly reduce their risk of vitamin D deficiency by taking a multivitamin tablet containing vitamin D.
